# The hMetrnl-PLGA-PEG-PLGA Hydrogel Facilitates Skin Wound Healing Through Dual Regulation on eNOS Activity and Stability

**DOI:** 10.3390/ph18081180

**Published:** 2025-08-10

**Authors:** Huan-Yu Zhao, Jie-Bing Jiang, Yu Chen, Chao-Yu Miao

**Affiliations:** 1 Department of Pharmacology, Second Military Medical University/Naval Medical University, Shanghai 200433, China; zhaohuanyu1128@smmu.edu.cn (H.-Y.Z.); jbjiang@smmu.edu (J.-B.J.); 2 Department of Gynecology and Obstetrics, The First Affiliated Hospital (Changhai Hospital), Second Military Medical University/Naval Medical University, Shanghai 200433, China; chenyu@smmu.edu.cn

**Keywords:** recombinant human Metrnl, eNOS, angiogenesis, wound healing, hMetrnl-PLGA-PEG-PLGA

## Abstract

**Background/Objectives:** Metrnl (Meteorin-like), a secreted protein identified in our lab, has been shown to promote wound healing in mice. However, current therapeutic strategies and the underlying mechanisms remain incompletely understood. This study aimed to (1) develop a recombinant human Metrnl (hMetrnl) hydrogel formulation for topical delivery, and (2) elucidate its molecular mechanism in wound repair. **Methods:** hMetrnl was dispersed in a thermosensitive PLGA-PEG-PLGA hydrogel (hMet-PPP) and applied topically to full-thickness skin wounds in male C57BL/6 mice. A large initial dose was administered on the day of injury, followed by a lower maintenance dose regimen. Mechanistic studies were performed using molecular/cellular assays to assess the effects of hMetrnl. **Results:** Administration of hMet-PPP significantly accelerated wound healing, reducing the initial wound area and shortening the overall recovery time. hMetrnl transmits signals to endothelial cells via the KIT receptor tyrosine kinase (C-Kit), a membrane receptor, thereby initiating a dual regulatory mechanism involving eNOS to promote angiogenesis: (1) rapid activation of eNOS activity within 30 min through the PI3K/AKT signaling pathway; and (2) suppression of proteasomal and lysosomal eNOS degradation, resulting in enhanced eNOS expression and prolonged functional activity under sustained treatment. **Conclusions:** Topical hMet-PPP administration represents a promising therapeutic strategy for enhancing early-stage wound healing. hMetrnl exerts its biological effects through C-Kit, which mediates dual regulation of eNOS, both activation and stabilization, providing a mechanistic basis for its potent angiogenic properties. These findings uncover a novel Metrnl mechanism with potential implications for the development of therapies targeting vascular dysfunction and tissue repair.

## 1. Introduction

Skins provide an integrated protective shield for bodies in defending against external injury, barring the entry of pathogens, and maintaining physiological homeostasis. When an injury occurs, the body initiates complex and effective repair mechanisms, which are broadly divided into four highly integrated and overlapping phases: (1) hemostasis, (2) inflammation, (3) proliferation, and (4) remodeling [1]. Current research suggests that the reduction of angiogenesis is a pivotal cause of non-healing wounds [2]. Angiogenesis plays a crucial role in wound healing through its complex regulations and spatiotemporal effects. It not only supplies the wound site with essential oxygen, nutrients, and bioactive substances but also removes debris and metabolic waste. Additionally, angiogenesis secretes multiple mediators that facilitate smooth wound healing [3,4,5].

Metrnl (also known as Meteorin-like), a secreted protein discovered in our lab, is involved in the regulation of various pathophysiological processes, such as maintenance of vascular homeostasis and protection against metabolic, inflammatory, and cardiovascular diseases [6,7,8,9,10]. In 2022, we first published the positive effects of Metrnl in angiogenesis and wound healing [11]. This showed that both Metrnl^KO/KO^ and endothelial cell-specific Metrnl gene knockout (Metrnl^loxP/loxP^Tek-Cre) mice significantly delay skin wound healing and impair VEGFA-mediated AKT/eNOS activation, thereby reducing angiogenesis [11]. Other researchers subsequently reported that keratinocyte- and M2 macrophage-derived Metrnl enhanced wound re-epithelialization and angiogenesis, and was beneficial to wound closure [12]. This fully reflects the value of exogenous Metrnl in the clinical application of skin injuries. However, how exogenous Metrnl governs the fate of endothelial cells and angiogenesis remains largely unclear. In addition, although Song et al. mixed recombinant Metrnl protein in Pluronic F-127 for facilitating wound healing in mice [12], more biocarriers and dosing strategies remain to be tested to perfect its application and improve the therapeutic value and efficiency. PLGA-PEG-PLGA thermosensitive hydrogel (PPP) is a triblock copolymer composed of Poly (d,l-lactide-co-glycolide)-poly (ethylene glycol)-poly (d,l-lactide-co-glycolide). It exhibits excellent temperature-responsive sol-gel phase transition behavior due to the corresponding differences in temperature between hydrophobic and hydrophilic structures, along with favorable biocompatibility and biodegradability [13,14,15]. Studies have shown that drugs can be loaded into the PLGA-PEG-PLGA hydrogel in the sol state at low temperature. Upon administration in vivo, the elevation of temperature to physiological levels triggers a phase transition into the gel state, enabling controlled drug release [16,17]. Therefore, PPP serves as a promising carrier for drug delivery [17,18,19]. It would be valuable to further investigate whether the combination of hMetrnl and PPP at the wound site can effectively promote wound healing.

We set out in this study to search for the therapeutic effects of exogenous recombinant human Metrnl (hMetrnl) dispersed in PPP, as well as the intracellular mechanism in promoting angiogenesis and wound healing. We show that the administration of hMetrnl-PPP (hMet-PPP) significantly reduces the initial wound area and accelerates the healing process. hMet-PPP activates eNOS and enhances the stability of its protein expression, collectively contributing to angiogenesis and wound repair. These findings confirm the beneficial role of exogenous Metrnl in promoting wound healing and angiogenesis. Combining hMetrnl with PPP offers a new therapeutic strategy. The molecular mechanism also provides a foundation for the investigation and treatment of other vascular diseases.

## 2. Results

### 2.1. Early High-Dose hMet-PPP Treatment Enhances Wound Healing in Mice

**Figure 1:**
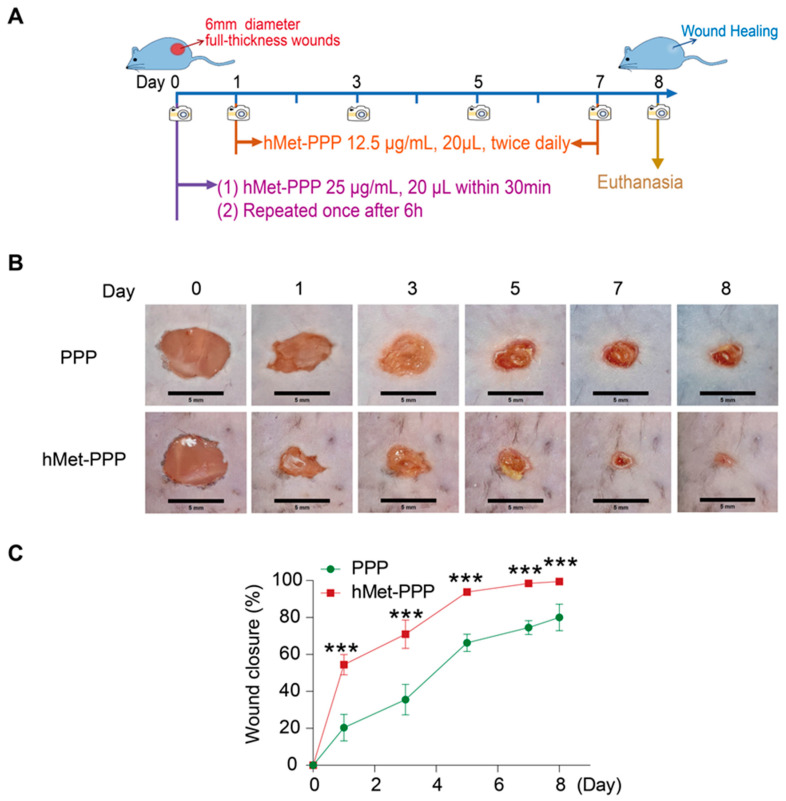
The administration of hMet-PPP promotes skin wound healing in mice. (**A**) The administration schedule for hMet-PPP or PPP on the day of injury and thereafter as indicated; (**B**) representative wound healing and (**C**) closure rates following PPP or hMet-PPP treatment at the designated time points (*n* = 6–10). Data in (**C**) are presented as mean ± SD. *** *p* < 0.001, as analyzed by two-way ANOVA.

We validated the therapeutic effect of hMetrnl in mice with full-thickness excisional wounds using a thermosensitive PLGA-PEG-PLGA (PPP) hydrogel formulation containing hMetrnl (hMet-PPP). The phase transition temperature of PPP is 30 ± 2 °C, enabling gel formation upon topical application and supporting localized, sustained release [20]. The treatment protocol involves local administration of hMet-PPP at a dose of 20 μL (25 μg/mL) within 30 min after injury, followed by a second dose 6 h later to complete the high-dose repeated regimen on the same day (Figure 1A). Subsequent doses are 20 μL of 12.5 μg/mL, given twice daily until wound closure is achieved (Figure 1A). Meanwhile, an equivalent volume of PPP was administered at the same time point as the control treatment. Overall, we found that the hMet-PPP treatment group exhibited a shorter duration required for skin wound healing compared to the control group (Figure 1B). By Day 8, most wounds in the treatment group had fully healed, while the control group showed approximately 78% healing (Figure 1C). This finding confirms the efficacy of the hMet-PPP formulation in accelerating wound healing. Specifically, administering a high dose (25 μg/mL, 20 μL within 30 min post-injury), followed by a repeat dose on the same day, resulted in a significant difference in wound healing rates observed on the following day. The control group exhibited approximately 20% healing, whereas the treatment group reached approximately 50%, nearly 2.5 times higher (Figure 1C, *p* < 0.001), indicating that increased dosing frequency and dosage positively influence early-stage wound healing.

### 2.2. hMetrnl Accelerates Neovascularization Around the Injured Tissues

**Figure 2:**
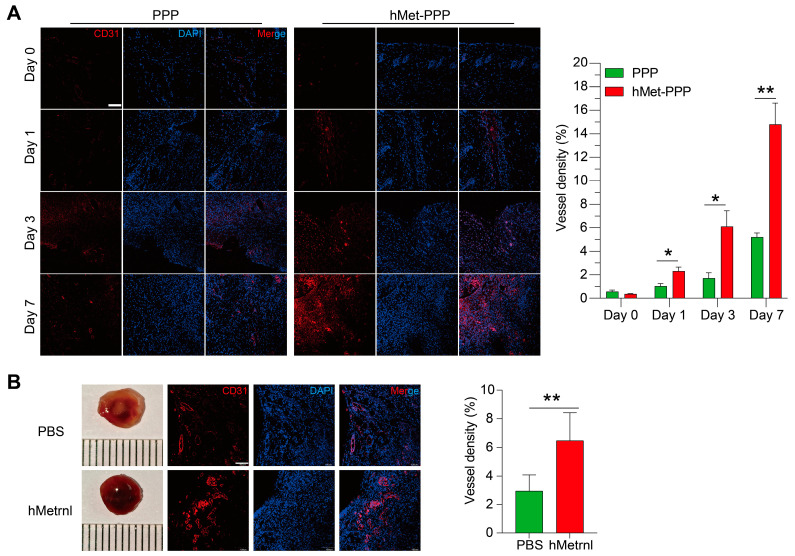
hMetrnl stimulates neovascularization. (**A**) Typical images of CD31 immunofluorescence-stained skin tissue at different time points, and quantitative analyses of CD31-positive areas on the sections (*n* = 3); (**B**) typical images of Matrigel plugs containing hMetrnl from mice (*n* = 8) and their sections immunofluorescence-stained, as well as quantitative analyses for CD31 (*n* = 5). Scale bar, 100 μm. Data are mean ± SD. * *p* < 0.05; ** *p* < 0.01, analyzed by two-tailed *t*-test (**A**) and Student’s *t*-test (**B**).

Fluorescent immunostaining of skin tissue surrounding the wound using the endothelial cell marker CD31 showed that repeated administration of hMet-PPP (25 μg/mL, 20 μL) increased vessel density by approximately 1.2-fold compared to PPP treatment alone on Day 1 (*p* < 0.05), with a more pronounced enhancement observed on Days 3 (*p* < 0.05) and 7 (*p* < 0.01) (Figure 2A). These suggest that hMet-PPP promotes angiogenesis in the surrounding tissue and accelerates wound healing. To confirm that hMetrnl, not PPP, is critical for the process, we employed a Matrigel plug angiogenesis model. We observed that Matrigel plugs containing hMetrnl appeared significantly redder upon subcutaneous extraction compared to control plugs without hMetrnl (Figure 2B). Consistently, blood vessel density was approximately twice as high in hMetrnl (300 ng/mL)-stimulated plugs as in PBS-treated plugs (Figure 2B, *p* < 0.01). These findings further support the role of exogenous Metrnl in promoting in vivo neovascularization.

### 2.3. Blocking C-Kit Inhibits hMetrnl-Facilitated Angiogenesis

**Figure 3:**
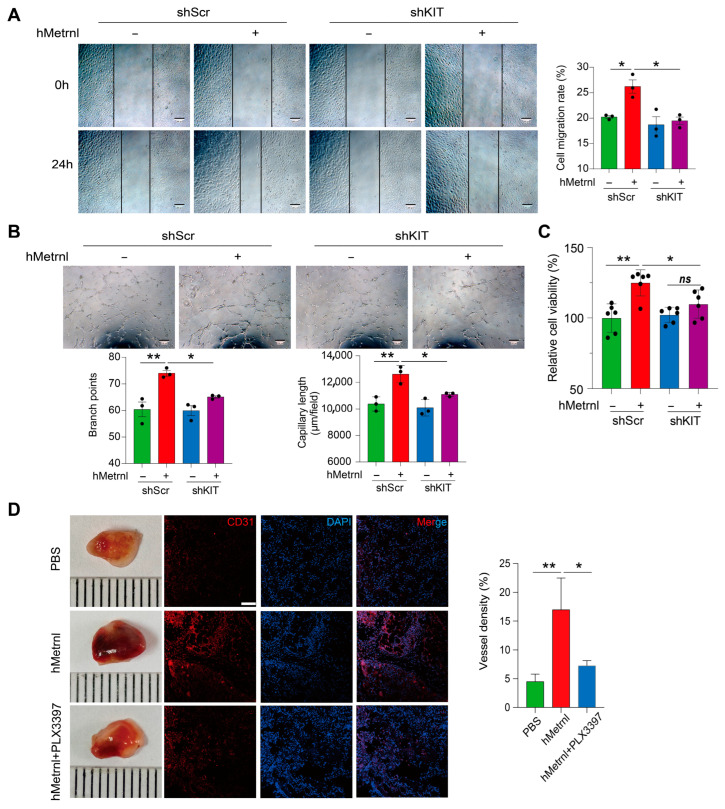
hMetrnl facilitates angiogenesis through the presence of KIT. (**A**) Typical images and quantification of primary HUVECs scratch wound healing treated with hMetrnl after transfection with shKIT or shScr (*n* = 3, scale bar, 200 μm); (**B**) typical microscopic photographs of capillary-like tubule structures formed by primary HUVECs, and quantitative analyses of the branch points and capillary lengths (*n* = 3, scale bar, 200 μm); (**C**) the proliferation abilities of primary HUVECs (*n* = 6); (**D**) typical images of Matrigel plugs treated with hMetrnl and C-Kit inhibitor PLX3397 retrieved from mice (*n* = 7); their sections immunofluorescence-stained for CD31 and quantitative analyses of vessel density (*n* = 3, scale bar, 100 μm). Data are mean ± SD. * *p* < 0.05; ** *p* < 0.01, analyzed by one-way ANOVA.

It is essential to explore the intracellular mechanisms of hMetrnl in facilitating neovascularization and wound healing. Reboll et al. reported that the stem cell factor receptor C-Kit is a high-affinity receptor for Metrnl in ischemic tissue repair [21]. Hence, we first determined whether silencing the KIT gene in HUVECs affects hMetrnl-induced angiogenesis. A scratch wound assay showed that hMetrnl treatment (100 ng/mL) increased cell migration in negative control lentivirus-transfected (shScr) HUVECs (Figure 3A, *p* < 0.05), consistent with our previous findings [11]. However, KIT gene knockdown obviously impaired this effect (Figure 3A). Similarly, hMetrnl treatment enhanced capillary-like tubule formation, branch number, and total length in shScr HUVECs, but had no effect in shKIT HUVECs. (Figure 3B). Furthermore, we found that hMetrnl increased shScr HUVEC proliferation by greater than 20% but failed in shKIT HUVECs (Figure 3C). These indicate that hMetrnl promotes angiogenesis in vitro, and KIT gene knockdown blocks this process. Similar results were observed in Matrigel plugs in vivo. The presence of both hMetrnl and PLX3397 (a selective C-Kit inhibitor) for gel plugs resulted in about a 40% reduction in the blood vessel density compared with that in the plugs with hMetrnl alone (Figure 3D, *p* < 0.05). Thus, C-Kit is an essential participant in hMetrnl-induced endothelial angiogenesis for transmitting signals into cells.

### 2.4. hMetrnl Initiates Intracellular PI3K/AKT/eNOS Signaling Cascade Activation

**Figure 4:**
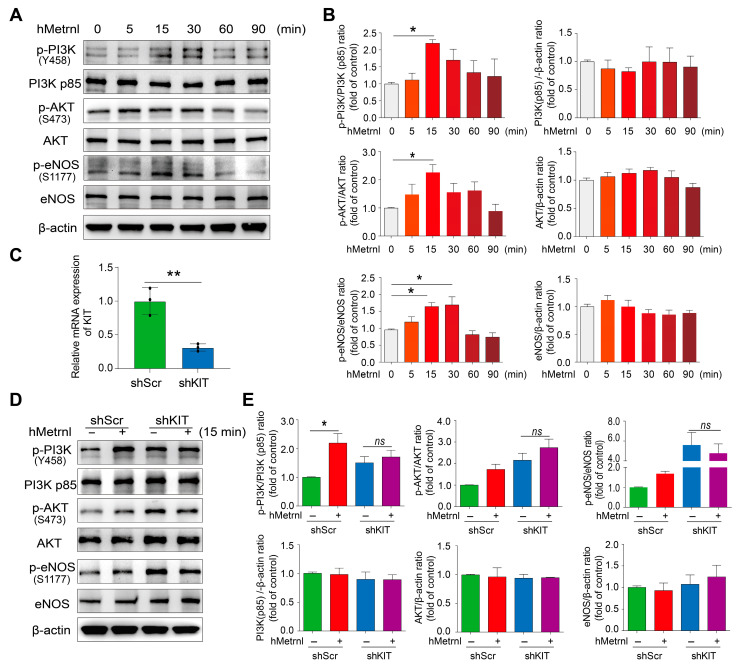
hMetrnl induces intracellular PI3K/AKT/eNOS activation within a brief time period. (**A**) The protein expression of p-PI3K, PI3K p85, p-AKT, AKT, p-eNOS, and eNOS in primary HUVECs with hMetrnl treatment for different times; (**B**) quantification of proteins expression of p-PI3K, PI3K p85, p-AKT, AKT, p-eNOS, and eNOS (*n* = 3); (**C**) mRNA levels of KIT in lentivirus-mediated KIT gene knockdown (shKIT) primary HUVECs (*n* = 3); (**D**) the protein expression of C-Kit, p-PI3K, PI3K p85, p-AKT, AKT, p-eNOS, and eNOS in primary HUVECs after shKIT/shScr transfection and treated hMetrnl for 15 min; (**E**) quantification of total and phosphorylated protein expression. Data are mean ± SD. * *p* < 0.05; ** *p* < 0.01, *n* = 3, data in (**C**) are analyzed by Student’s *t*-test, and others by one-way ANOVA.

C-Kit dimerizes when its classical ligand, stem cell factor (SCF), binds to the ectodomain, triggering conformational changes that enable lateral interactions between the D4 and D5 domains of adjacent receptors [22]. These changes subsequently lead to autophosphorylation, which promotes the conversion of Ras-GDP to active Ras-GTP, thereby activating downstream signaling pathways such as PI3K, MAPK, and Raf [23]. After 15 min of hMetrnl treatment, the phosphorylation levels of PI3K p85 and AKT in primary HUVECs increased by approximately 120% (*p* < 0.05), and phosphorylated eNOS increased by 1.7-fold (*p* < 0.05) (Figure 4A,B). In contrast, the total protein expression levels of these molecules remained unchanged (Figure 4A,B). Activated eNOS plays a critical role in stimulating endothelial nitric oxide production and maintaining signal strength, thereby regulating multiple vascular homeostasis signals, including angiogenesis [24]. The activation of eNOS (phospho-eNOS) could still be detected after 30 min (Figure 4A,B). These suggest that hMetrnl rapidly activates eNOS via the intracellular PI3K-AKT-eNOS signaling cascade, thereby accelerating angiogenesis. To further verify whether the activation of the above signaling is mediated by C-Kit receptor, we generated shKIT HUVECs that reduced C-Kit expression by approximately 80% (Figure 4C,D). In these cells, 15 min hMetrnl treatment failed to increase eNOS phosphorylation or activate the PI3K-AKT pathway (Figure 4D,E). Therefore, C-Kit is the gate for hMetrnl to transmit the action into cells and then rapidly activate eNOS.

### 2.5. hMetrnl Enhances eNOS Protein Stability by Inhibiting Degradation

**Figure 5:**
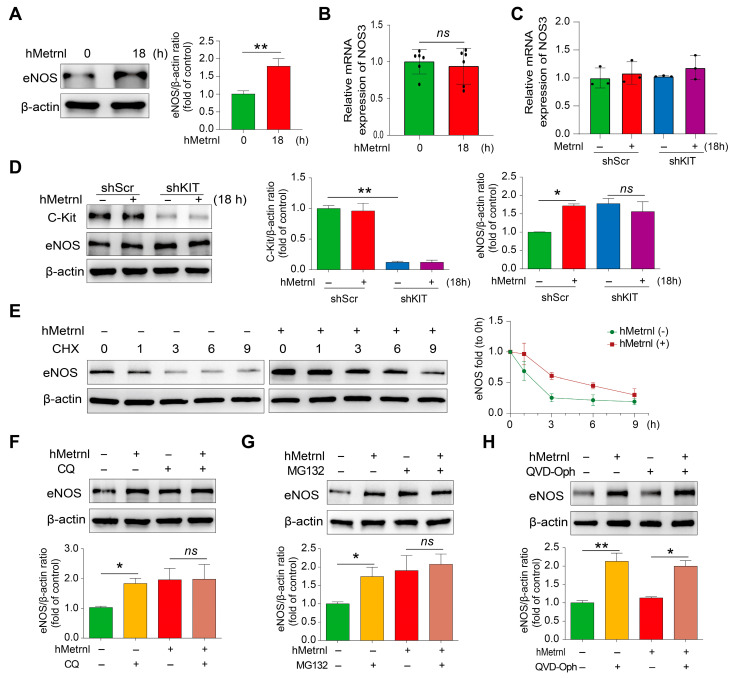
hMetrnl suppresses eNOS reduction via impacting lysosome and protease degradation pathways. (**A**,**B**) The protein expression and mRNA levels of eNOS (NOS3) in primary HUVECs with hMetrnl treatment for 18 h; (**C**) mRNA levels of NOS3 in shKIT primary HUVECs with hMetrnl treatment for 18 h; (**D**) the protein expression and quantitative analyses of C-Kit and eNOS; (**E**) half-life analysis of eNOS with hMetrnl treatment in primary HUVECs by CHX (100 μM), and the degradation rate curve of eNOS; (**F**–**H**) the protein levels of eNOS and quantification analysis in primary HUVECs treated with the inhibitors targeting lysosome (CQ), proteasome (MG132), and pan-caspase (QVD-Oph) in the presence of hMetrnl administration. Data are mean ± SD. * *p* < 0.05; ** *p* < 0.01, *n* = 3; data in (**A**,**B**) are analyzed by Student’s *t*-test, and others by one-way ANOVA.

Studies have demonstrated that impaired wound healing is associated with reduced levels of eNOS [25]; in contrast, upregulation of eNOS expression promotes angiogenesis and enhances cutaneous wound healing [26]. First, we observed that hMetrnl treatment for 18 h increased eNOS protein expression by approximately 1.7-fold compared to the unstimulated group (Figure 5A, *p* < 0.01). However, no significant change in NOS3 mRNA expression was detected after hMetrnl treatment (Figure 5B). Consistently, hMetrnl had no effect on NOS3 mRNA levels in KIT knockdown HUVECs (Figure 5C); nevertheless, the absence of KIT blocked the hMetrnl-induced increase in eNOS protein expression (Figure 5D), indicating that hMetrnl regulates eNOS expression through the C-Kit receptor at the translational or post-translational level. Second, the cycloheximide (CHX) assay revealed that hMetrnl treatment significantly slowed the eNOS degradation compared to the control group (Figure 5E), suggesting that reduced degradation may contribute to elevated eNOS levels. To investigate the underlying mechanism, we used chloroquine (CQ), MG132, and QVD-Oph, the specific inhibitors of lysosomal, proteasomal, and pan-caspase pathways, respectively. Results showed that both CQ and MG132 significantly increased eNOS expression (*p* < 0.05 compared to control group), and this effect was not further enhanced by hMetrnl (Figure 5F,G). In contrast, QVD-Oph neither influenced basal eNOS expression nor suppressed hMetrnl-induced eNOS upregulation (Figure 5H). These findings indicate that hMetrnl enhances eNOS stability by inhibiting lysosomal and proteasomal degradation pathways that upregulate expression.

### 2.6. hMet-PPP Accelerates Wound Healing Through eNOS Regulation Mechanism

**Figure 6:**
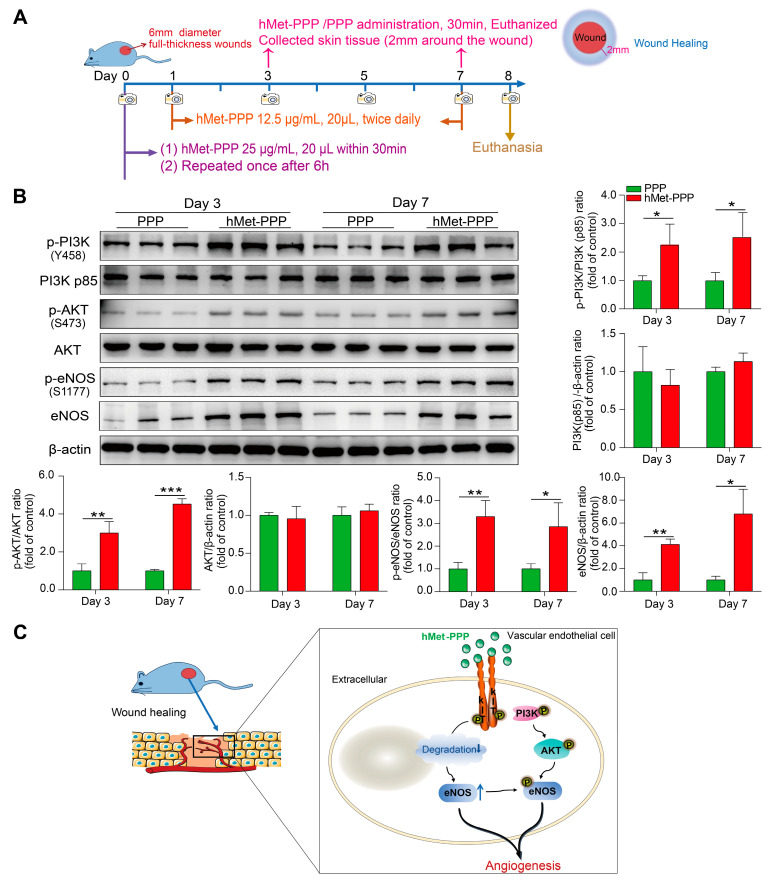
hMet-PPP modulates the activity and expression of eNOS in the perilesional skin tissues. (**A**) Experimental design and timeline for modeling, hMet-PPP or PPP administration, and sample collection; (**B**) the protein expression and quantitative analyses of p-PI3K, PI3K p85, p-AKT, AKT, p-eNOS, and eNOS in skin tissue around 2 mm of the wound at Day 3 and Day 7 (three samples per group; experiment repeated once); (**C**) schematic representation of molecular mechanisms by which hMetrnl facilitates angiogenesis. Data are mean ± SD. * *p* < 0.05; ** *p* < 0.01; *** *p* < 0.001, *n* = 3, analyzed by Student’s *t*-test.

We subsequently investigated whether the eNOS-related mechanisms were present at the wound site. Mice were euthanized 30 min after administration of hMet-PPP or PPP on Days 3 and 7, and skin tissues within approximately 2 mm of the wound margin were collected (Figure 6A). Our results showed that the phosphorylation levels of PI3K, AKT, and eNOS were significantly increased on both Day 3 and Day 7 in the hMet-PPP-treated group (Figure 6B; *p* values: Day 3—*p* < 0.05, *p* < 0.01, and *p* < 0.01; Day 7—*p* < 0.05, *p* < 0.01, and *p* < 0.05). This indicates that the PI3K/AKT/eNOS signaling pathway was activated within 30 min after hMet-PPP treatment, which is consistent with the in vitro findings. Furthermore, hMet-PPP treatment also increased eNOS total protein expression (Figure 6B, Day 3—*p* < 0.01; Day 7—*p* < 0.05 vs. control group). These findings further confirm that hMetrnl activates the PI3K/AKT/eNOS signaling pathway during short-term exposure and upregulates eNOS protein expression following long-term treatment, which aligns with the observed in vitro mechanisms.

## 3. Discussion

We demonstrate in this study that hMet-PPP effectively accelerates skin wound healing in mice by exerting a dual regulatory effect on intracellular eNOS, thereby promoting angiogenesis. Specifically, the administration of hMet-PPP, involving an initial large dose (0.5 μg/mL, 20 μL) twice on the day of injury followed by a reduced maintenance dosing (0.25 μg/mL, 20 μL) regimen, significantly reduces the initial wound area and accelerates the healing process. The underlying mechanism suggests that hMetrnl signals intracellularly via C-kit, rapidly activates eNOS through the PI3K/AKT signaling cascade within a short timeframe, and stabilizes eNOS by inhibiting lysosomal and proteasomal degradation pathways, thereby elevating protein levels and sustaining its prolonged effects (Figure 6C).

The thermosensitive PPP hydrogel (30 ± 2 °C) was in a liquid flow state at low temperature, allowing hMetrnl to dissolve and disperse easily, thereby forming hMet-PPP as a topical formulation for wound treatment. Once hMet-PPP is applied to the wound, it undergoes gelation, a process that not only facilitates the prolonged release of hMetrnl for a long-lasting effect but also prevents direct contact between the wound and the external environment [18,27]. Similarly, Song et al. utilized Pluronic F127 as a carrier to encapsulate recombinant mouse Metrnl, thereby effectively enhancing wound healing in diabetic mice [12]. This is consistent with our overall findings that exogenous Metrnl treatment accelerates wound healing, although there are some differences, including the selection of hydrogel, the species of recombinant Metrnl protein used, the dosage administered, and the administration regimen. Pluronic F127 is also an excellent thermosensitive hydrogel that serves as a delivery system for various beneficial substances to promote wound healing [28,29]. The dosage they used was 60 μg twice daily, which is approximately 120 times as high as our highest dose. The significant differences may be attributed to the different sources of Metrnl and the distinct hydrogel selected. Additionally, we adopted a strategy of double-dose administration during the early stage of wound healing, followed by maintenance with a single dose, which differs from their fixed dosing regimen. Therefore, we not only demonstrated the therapeutic value of low-concentration hMet-PPP in promoting wound healing but also confirmed the beneficial effect of rapid and early double-dose administration following injury in accelerating the healing process. These findings have significant implications for the selection of appropriate dosing regimens in hMetrnl-based treatment strategies. Furthermore, Metrnl secreted by surrounding skin tissue cells, such as quiescent fibroblasts, keratinocytes, and immune cells, may also synergistically accelerate angiogenesis and wound healing, such as resting fibroblasts, keratinocytes, and immune cells [21,30].

Reboll et al. first reported in 2022 that Metrnl is a high-affinity ligand for C-Kit receptor in the context of ischemic tissue [21]. We observed that C-Kit deficiency hindered Metrnl-induced angiogenesis in mouse subcutaneous Matrigel implants and cultured endothelial cells. Therefore, it is suggested that the C-kit receptor is involved in the intracellular signaling regulated by exogenous Metrnl. SCF is the classical ligand of the C-Kit receptor. Its mechanism of action is well established: it binds to the extracellular domain of C-Kit, promoting dimerization and subsequent autophosphorylation, thereby activating downstream intracellular signaling pathways. However, how Metrnl signals through C-Kit remains unclear. It is unknown whether this occurs through direct ligand activation or the involvement of other molecules. When the signal enters the cell, the “dual regulation” intracellular mechanism identified in this study may offer a spatiotemporally precise regulatory framework to mediate hMetrnl-stimulated angiogenic and wound healing. eNOS was first rapidly activated via the PI3K/AKT signaling cascade. eNOS, an endothelial-cell-specific isoform of the nitric oxide-producing enzyme, exerts precise regulation over crucial endothelial functions, such as maintaining vasodilation and supporting cardiovascular homeostasis [31], and it also plays a predominant role in both angiogenesis and vasculogenesis [32]. The synthesis of nitric oxide, along with the intensity and duration of its signaling effects, is predominantly regulated by eNOS activity. Thus, rapid activation of eNOS in endothelial cells at the wound site modulates injury-induced endothelial dysfunction and accelerates angiogenesis. However, activation of the PI3K/AKT/eNOS signaling cascade exhibits a rapid yet transient nature. Notably, in addition to inducing eNOS activation, prolonged treatment with hMetrnl significantly impacted eNOS degradation, leading to upregulation of eNOS protein expression. It has been reported that increased eNOS expression promotes angiogenesis and enhances skin wound healing in mice [26]. The transcription or protein expression of eNOS was moderately upregulated during the physiological process of skin injury repair. Furthermore, we observed that knockdown of the KIT gene alone robustly activated the PI3K/AKT/eNOS signaling pathway, suggesting potential involvement of the KIT gene itself with other mechanisms of this signal cascade. Collectively, the hMetrnl/C-Kit exerts a dual regulatory function in endothelial cells by modulating both eNOS activity and protein stability levels (Figure 6C).

The present findings not only elucidate for the first time how Metrnl promotes angiogenesis through dual modulation of eNOS but also propose hMet-PPP as a promising therapeutic strategy for enhancing neovascularization and wound repair. However, certain limitations and unresolved mechanisms remain to be addressed. A limitation of the hMet-PPP formulation is its requirement for low-temperature dispensing and storage prior to administration, which may be relatively inconvenient. Whether it can remain stable in a gel state at room temperature remains unclear. Further experiments are needed to evaluate the failure timing and the impact on Metrnl activity and release. Alternatively, further optimization of the formulation is warranted to improve its practicality and ease of use. In addition, the hMet-PPP formulation could be combined with supplementary components, such as polysaccharides [33], oils [34], and advanced wound dressings [35], to synergistically enhance the wound healing process. Mechanistically, although Metrnl is currently established as a high-affinity ligand of C-Kit, it remains unclear whether hMetrnl, like SCF, functions as a direct ligand for C-Kit activation or whether other molecules are involved. Moreover, the mechanism by which Metrnl prevents lysosomal and proteasomal degradation of eNOS is still unclear. Therefore, future research might focus on two aspects: optimizing therapeutic formulations and elucidating the gaps in the mechanism in order to better understand the in vivo functions of the secreted protein Metrnl and enhance its translational potential.

## 4. Materials and Methods

### 4.1. Cell Culture and Treatment

The primary human umbilical cord vein endothelial cells (HUVECs) used in this study were isolated and pooled from freshly delivered umbilical cords in our laboratory using previously established methods [36]. HUVECs were cultured in endothelial cell medium (ECM) (Sciencell, Carlsbad, CA, USA) supplemented with 5% fetal bovine serum (FBS), 1% endothelial cell growth supplement (ECGS), and 1% penicillin–streptomycin (PS). The mouse endothelial cell line bEnd.3 was purchased from the American Type Culture Collection (ATCC, Rockefeller, VI, USA) and maintained in DMEM (Invitrogen, Carlsbad, CA, USA) containing 10% FBS (Gibco, Carlsbad, CA, USA) and 1% PS (100 U/mL penicillin, 0.1 mg/mL streptomycin) (Invitrogen, Carlsbad, CA, USA). All cells were cultured with 5% CO_2_ at 37 °C.

For lentivirus-mediated KIT gene knockdown, we commissioned Hanhang Science and Technology Co., Ltd. (Shanghai, China) to construct three HBLV-h-KIT shRNA (shKIT) vectors using pHBLV-U6-MCS-PGK-PURO as the interference vector. We subsequently screened these constructs to identify the one with the highest knockdown efficiency in HUVECs for further studies. The selected shKIT sequence was TATCAGTTCAGCGAGAGTTAA.

Cells were treated with 100 ng/mL hMetrnl (R&D Systems, MN, USA) for varying durations or pre-treated with 50 μM chloroquine (CQ, Selleck, Houston, TX, USA), 10 μM MG132 (MedChemexpress, NJ, USA), or 10 μM Q-VD-OPh (MedMol, Shanghai, China) for 2 h prior to co-treatment with hMetrnl.

### 4.2. Animals and Wound Closure Assay

Male C57BL/6 mice (6–8 weeks old) were obtained from Ji Hui Experimental Animal Breeding Co., Ltd. (Shanghai, China) for wound closure assay and angiogenesis studies and raised and handled under specific pathogen-free (SPF) conditions. Each mouse is designated as an individual experimental unit. The details of experimental protocols were submitted to the Medical Research Ethics Committee of the Naval Medical University prior to the initiation of the study. All animal care and laboratory procedures were approved by the Institutional Animal Care and Use Committee of Naval Medical University (Shanghai, China) and conformed to the Institutional Animal Care Guidelines. Anesthesia was induced with isoflurane, followed by rapid cervical dislocation after the mouse had fully lost consciousness, thereby ensuring humane and effective euthanasia.

For skin wound experiments, mice were anesthetized with isoflurane, and full-thickness wounds were created on their backs using a sterile 6 mm round biopsy punch to fully simulate human skin wounds. The day of wounding was designated as Day 0, and the wounds were photographed for documentation. After each mouse was numbered in a single cage, all animal units were randomly assigned to experimental groups using a random number table. Blind methodologies were employed throughout the experimental and testing procedures. Hydrogel preparation, animal grouping, and data analysis were performed by separate research personnel to ensure objectivity. Within 30 min post-wounding, the wounds were topically treated with either hMetrnl-loaded PLGA-PEG-PLGA hydrogel (hMet-PPP) or PLGA-PEG-PLGA hydrogel (PPP) alone as the control group. The PLGA-PEG-PLGA (30 ± 2 °C) hydrogel was purchased from Daigang biomaterial Co., Ltd (Jinan, China) and prepared by dissolving in sterile water at a ratio of 1:5 to form a transparent liquid. The prepared hydrogel exhibits temperature-sensitive properties; specifically, it remains in a flowing liquid state at 4 °C, which facilitates the incorporation of hMetrnl to form hMet-PPP. Upon reaching approximately 30 °C, it transitions into a gel-like state. This temperature (30 ± 2 °C) falls well within the range of wound surface temperatures, enabling rapid gel formation at the wound site and thereby delaying the release of hMetrnl. hMetrnl was dissolved in this hydrogel solution at 25 μg/mL and 12.5 μg/mL and administered according to different predetermined protocols until complete wound healing was achieved. The protocol involved administering 20 μL of 25 μg/mL hMet-PPP within 30 min after injury, followed by a second dose 6 h later. A maintenance dose of 20 μL of 12.5 μg/mL was administered twice daily, starting on Day 1 and continuing until complete wound healing was observed. Photographs were taken on Day 0, on subsequent odd-numbered days (e.g., 1, 3, 5, and 7), and on the day of wound healing to calculate the rate of wound closure [11]. The fully healed mice were euthanized at the designated humane endpoint. Immediately following total skin excision on Day 0 and 30 min after administration of hMet-PPP or PPP on Days 1, 3, and 7, three mice (*n* = 3) from each group were randomly selected. Skin tissue samples collected from within 2 mm of the wound margin following euthanasia were subjected to subsequent immunofluorescence analysis. Likewise, an additional three samples (*n* = 3) per group were randomly selected on Day 3 and Day 7 for Western blot detection. No unexpected incidents occurred among all the mice during the study period, and all results from these samples were included in the statistical analysis, with no animals or data points excluded. The control group (PPP) and the treatment group (hMet-PPP) each comprised 24 experimental units, with a total of 48 mice/units used. This sample size was determined based on the number of mice utilized for post-euthanasia testing at predefined midpoint time points during the treatment period, as well as the final confirmation of six mice exhibiting complete healing.

Wound closure rate (%) = (Day 0 wound area − Day X wound area)/Day 0 wound area × 100%. The wound area on Day X represents the skin wound area at various time points following model establishment.

### 4.3. Angiogenesis Assay

A method of in vivo Matrigel plug angiogenesis assay [37] was adopted. First, bEnd.3 cells underwent hMetrnl (100 ng/mL) treatment with/without 0.5 μM Pexidartinib (PLX-3397, a selective inhibitor of C-Kit) in vitro for 18 h, then the cells were added to the Matrigel (catalog # 356231, Corning, Corning, NY, USA) to prepare a 5 × 10^6^/mL suspension containing hMetrnl (300 ng/mL) with/without PLX-3397 (0.5 μM), and shaken gently for 4 h at 4 °C. The cell suspension (200 μL) was injected subcutaneously into the abdomen of male C57BL/6J mice and solidified at body temperature. A single mouse was an experimental unit. It is marked as Day 0. Then, 60 ng hMetrnl (300 ng/mL) with/without PLX3397 (0.5 μM) was injected in situ on Days 2, 4, and 6. The mice were euthanized, and the gel plugs were retrieved on the 7th day for photos. Three units (*n* = 3) were randomly selected from each group for immunofluorescence analysis. Protocol 1 (Figure 2B): The control group (PBS) and the treatment group (hMetrnl) each comprised 8 experimental units, with a total of 16 mice/units used. Protocol 2 (Figure 3D): Each of the control, treatment, and inhibitor groups contained 7 experimental units, resulting in a total of 21 mice/units used. No unexpected incidents occurred among all the mice during the study period, and all results from these samples were included in the statistical analysis, with no animals or data points excluded. All animal units were randomly assigned to experimental groups using a random number table, and each group was housed in 1–2 cages (3–4 mice per cage). Blind methodologies were implemented throughout the experimental and testing procedures. Matrigel preparation, animal grouping, and data analysis were conducted by distinct research personnel to ensure objectivity.

### 4.4. Tube Formation Assay

The in vivo Matrigel plug angiogenesis assay was conducted as previously described [37]. Lentivirus-transfected HUVECs were pretreated with hMetrnl for 18 h and subsequently re-seeded into a 48-well plate (2 × 10^4^ cells per well) pre-coated with 200 μL of Matrigel (catalog # 356231, Corning). After incubation at 37 °C for 4 h, the cells were imaged using an Olympus CKX41SF microscope. The length and number of branch points of the capillary-like structures were quantified using Image-J software V1.54 (NIH, Bethesda, MD, USA).

### 4.5. Immunofluorescence Staining (IF)

The skin tissue excised from within a 2 mm area surrounding the wound was carefully placed onto a slide. After adding an appropriate amount of 4% paraformaldehyde on the tissue, a coverslip was applied to flatten the tissue. The resulting sandwich-like assembly was then immediately submerged completely in 4% paraformaldehyde solution for fixation. Matrigel plugs were placed directly into a 4% paraformaldehyde solution for fixation immediately after their removal. Fresh skin tissues and Matrigel plugs were embedded in paraffin and sectioned at a thickness of 4 μm. The paraffin sections were deparaffinized, rehydrated, and subjected to antigen retrieval using sodium citrate buffer. Before incubation of the primary antibody, it was blocked with 10% donkey serum for 1 h to reduce nonspecific binding. Immunostaining was performed sequentially: first with the primary antibody (CD31,1:100 catalog #AF3628, R&D Systems) overnight at 4 °C, followed by incubation with an Alexa Fluor 488-conjugated secondary antibody (catalog # A-11055, Invitrogen) for 90 min at room temperature. Nuclei were counterstained with DAPI (Beyotime, Beijing, China) for 10 min. Images were acquired using a laser confocal microscope (FV3000, Olympus, Tokyo, Japan). Vessel density was quantified using Image-J software.

### 4.6. Scratch Wound Assay

HUVECs transfected with lentivirus were prepared in advance. Horizontal lines were drawn on the back of the six-well plate at equal distances above and below the center of each well. Lentivirus-transfected HUVECs were then seeded into these plates and cultured to 100% confluence under hMetrnl (100 ng/mL) or PBS stimulation. The monolayers were subsequently scratched using a 200 μL pipette tip, perpendicular to the previously drawn line. Debris from the scratch was removed by washing with PBS, and images were captured at the intersection points. After 24 h of continued culture in ECM medium without FBS, cells were re-imaged at the same locations in the presence of hMetrnl or PBS. Images were acquired using an inverted microscope (CKX41SF, Olympus, Tokyo, Japan). Cell migration rates were analyzed by Image-J software.

### 4.7. Proliferation Analysis

Cell proliferation was assessed using the Cell Counting Kit-8 (CCK-8) assay. Lentivirus-transfected HUVECs were seeded in 96-well plates at a density of 1 × 10^3^ cells per well and starved for 12 h after attachment. Subsequently, the cells were treated with hMetrnl or PBS in ECM complete medium for 24 h. The medium was then replaced with 100 μL of CCK-8 mixed solution (Beyotime), and the plates were incubated at 37 °C for 3 h. Absorbance was measured at 450 nm using an ELISA reader (K3 Labserv, Thermo Fisher, Waltham, MA, USA).

### 4.8. RNA Isolation and Real-Time qPCR

Total RNA was isolated and extracted using the SteadyPure Universal RNA Extraction Kit II (Accurate Biology, Hunan, China). Reverse transcription and quantitative real-time PCR were performed by the Evo M-MLV RT Premix kit and the Green Premix pro Taq HS qPCR kit (Accurate Biology), respectively. The primer sequences were as follows: h-KIT, 5′-GGCACGGTTGAATGTAAGGC and 5′-AGGGTGTGGGGATGGATTTG; h-eNOS, 5′-GTGGCTGGTACATGAGCACT and 5′-GTCTTTCCACAGGGACGAGG; and h-GAPDH, 5′-GGAGCGAGATCCCTCCAAAAT and 5′-GGCTGTTGTCATACTTCTCATGG.

### 4.9. Western Blot Analysis

RIPA Lysis Buffer supplemented with 1% PMSF and 1% protease inhibitor cocktail (Beyotime) was utilized to extract total protein from both tissue and cell samples. The tissue sample consisted of skin tissue collected from the area within 2 mm surrounding the wound. Following the addition of lysis buffer, the tissue was homogenized three times using a pre-chilled tissue homogenizer, with a 5 min interval between each homogenization cycle to ensure complete lysis. The supernatant was collected after centrifugation for concentration determination. It was quantified using the bicinchoninic acid (BCA) protein assay kit (Beyotime). Subsequently, equal amounts of protein were separated by SDS-PAGE electrophoresis, transferred onto PVDF membranes (Millipore, Bedford, MA, USA), and blocked with Blocking Buffer (Beyotime) for 1 h at room temperature. The membranes were then incubated overnight at 4 °C with primary antibodies against Phospho-c-Kit (Tyr719), c-Kit, eNOS, Phospho-eNOS (Ser1199), PI3K p85, Phospho- PI3K p85 (Tyr458), AKT, Phospho-AKT (Ser473) (all from Cell Signaling Technology, Mass, Danvers, MA, USA), or β-actin (Proteintech, Wuhan, China). After washing, the membranes were incubated with appropriate horseradish peroxidase-conjugated secondary antibodies (Beyotime). Finally, the blots were exposed to ECL Western blot reagents (Beyotime) and visualized with a chemiluminescence imaging system (Tanon, Shanghai, China).

### 4.10. Statistical Analysis

Statistical analyses were performed as mean ± SD using GraphPad Prism 9.0, which automatically calculates the confidence interval (GraphPad Prism Software, San Diego, CA, USA). Data normality was assessed using the Shapiro–Wilk test, and all results in this study satisfied the assumption of normality. Student’s *t*-test, one-way ANOVA, and two-way ANOVA were employed for comparisons between two groups, multiple groups, and multiple time points, respectively. Statistical differences with *p* < 0.05 were considered significant.

## Data Availability

All data from the study are included in the manuscript.
